# P^3^OI-MELSH: Privacy Protection Target Point of Interest Recommendation Algorithm Based on Multi-Exploring Locality Sensitive Hashing

**DOI:** 10.3389/fnbot.2021.660304

**Published:** 2021-04-23

**Authors:** Desheng Liu, Linna Shan, Lei Wang, Shoulin Yin, Hui Wang, Chaoyang Wang

**Affiliations:** ^1^College of Information and Electronic Technology, Jiamusi University, Jimusi, China; ^2^Software College, Shenyang Normal University, Shenyang, China

**Keywords:** point of interest, privacy protection, multi-exploring locality sensitive hashing, homomorphic encryption, location-based social network (LBSN)

## Abstract

With the rapid development of social network, intelligent terminal and automatic positioning technology, location-based social network (LBSN) service has become an important and valuable application. Point of interest (POI) recommendation is an important content in LBSN, which aims to recommend new locations of interest for users. It can not only alleviate the information overload problem faced by users in the era of big data, improve user experience, but also help merchants quickly find target users and achieve accurate marketing. Most of the works are based on users' check-in history and social network data to model users' personalized preferences for interest points, and recommend interest points through collaborative filtering and other recommendation technologies. However, in the check-in history, the multi-source heterogeneous information (including the position, category, popularity, social, reviews) describes user activity from different aspects which hides people's life style and personal preference. However, the above methods do not fully consider these factors' combined action. Considering the data privacy, it is difficult for individuals to share data with others with similar preferences. In this paper, we propose a privacy protection point of interest recommendation algorithm based on multi-exploring locality sensitive hashing (LSH). This algorithm studies the POI recommendation problem under distributed system. This paper introduces a multi-exploring method to improve the LSH algorithm. On the one hand, it reduces the number of hash tables to decrease the memory overhead; On the other hand, the retrieval range on each hash table is increased to reduce the time retrieval overhead. Meanwhile, the retrieval quality is similar to the original algorithm. The proposed method uses modified LSH and homomorphic encryption technology to assist POI recommendation which can ensure the accuracy, privacy and efficiency of the recommendation algorithm, and it verifies feasibility through experiments on real data sets. In terms of root mean square error (RMSE), mean absolute error (MAE) and running time, the proposed method has a competitive advantage.

## Introduction

Personalized recommendation system actively provides users with information services that best meet their interests by capturing the users' behavior preferences and information requirements (Sang et al., 2006). At present, personalized recommendation system has been successfully applied in social network, e-commerce and other mainstream Web services, such as Netflix, Amazon, Taobao, etc., and has been increasingly concerned and studied in various fields. Wireless communication technology has sustainably been developed. Location-based services (LBS) have attracted increasing attention, many users share their information on social applications (Jing et al., 2019; Yang et al., 2019). In location-based social network (LBSN), it becomes an important research to conduct point of interest (POI) recommendation based on the check-in data of users (Li, 2015; Xindi et al., 2019). Check-in data reflect users' preferences for locations, which provides a solid basis for personalized POI recommendation. This recommendation method not only allows users to search new relevant places without spending much time, but also allows service providers to provide accurate recommendation services to users.

Although the recommendation system has been studied in the past decades, recently, personalized POI recommendation (Luan et al., 2017; Zhu et al., 2019) has attracted people's attention because of its characteristics. Compared with the user-item scoring matrix in traditional recommendation system, the user-check-in matrix in POI recommendation is usually sparser. The sparsity of the user check-in data makes it difficult for the recommendation system to capture the users' preference for the location. Geography is another factor that distinguishes POI recommendations from traditional items. Analysis of check-in data shows that users tend to move from near to far. Besides, the user's history check-in information is often local and dense, which makes the “cold start” problem more prominent in the POI recommendation (Zhang et al., 2017). That's because even if a user has visited enough places near where they live when they go to new places, they will inevitably run into the “cold start” problem.

To solve the problem of sparse matrix and cold start, POI recommendation can be made by collecting data from multiple distributed platforms (Poutanen et al., 2017). For example, user A has check-in data on Weibo, and user B has check-in data on Facebook. User A combines the check-in data on Weibo and Facebook to make POI recommendation. But in this case, there are two big challenges. On the one hand, due to data privacy issues, Weibo and Facebook are reluctant to disclose their internal user check-in data to each other, which makes it difficult to calculate the similarity between user A and user B before making further recommendation. On the other hand, user check-in data is distributed in multiple platforms, which inevitably requires message communication between platforms, this consumes a lot of time and cannot meet users' requirements in terms of quick response. Therefore, we propose a P^3^OI (privacy protection point of interest) recommendation algorithm based on multi-exploring locality sensitive hashing. Similar user sets are selected by modified LSH, which can greatly reduce computation and satisfy users' demand for quick response. The LSH is improved by introducing multi-exploring to relieve the pressure of multiple hash tables on memory, and quickly get the set of target users' nearest neighbors. Modified LSH and Paillier homomorphic encryption technology are utilized in the calculation process to protect data privacy. Experimental results on real data sets show that the proposed algorithm is superior to the traditional user-based collaborative filtering recommendation algorithm and the state-of-the-art algorithms in terms of response time and prediction accuracy.

The remainder of this paper is organized as follows. Section “Related Works” discusses the related work on the recommendation system and POI research actuality. Section “Proposed P^3^OI Recommendation Algorithm” briefly analyzes the relevant definitions used for our proposed algorithm in this paper and discusses the implementation of the proposed algorithm in detail. Section “Experiments and Analysis” evaluates the experimental results and gives the analysis. Finally, section “Conclusion” makes a conclusion for this paper and outlines our future works.

## Related Works

Collaborative filtering is one of the most widely used technologies in the current recommendation system. Different from the content-based recommendation system, the collaborative filtering recommendation is based on the project score. It does not need to consider the project, so it is not limited by content. The recommended effects are diverse and novel. The collaborative filtering recommendation system is based on the behavioral analysis of group users or the measurement of interests similarity. By collecting users' comments on information or other behaviors, it searches for users with similar interests. Then, recommendation results are generated to the current users based on similar users' comments on other information.

Collaborative filtering is a widely used technique in recommendation systems. There are two types of collaborative filtering: memory-based collaborative filtering and model-based collaborative filtering (Ortega and Hernando, 2012; Bilge et al., 2014).

Memory-based collaborative filtering can be divided into user-based and item-based collaborative filtering. For a user-based system, the similarity between all users is calculated based on their ratings of related projects, which is calculated by the similarity measurement method. The score of missing items are then weighted by similar users for the same item. For the item-based system, similar scoring items are found and user scores of similar items are used to make prediction (Sunitha and Adilakshmi, 2014). However, memory-based collaborative filtering makes recommendations based on a collection of user preferences for items. The idea behind this approach is that active users' interests are more likely to align with those of users with similar preferences. Hence, the choice and computation of a similarity measure between users is critical to rating items.

In user-based collaborative filtering, user relationship groups can be discovered through similarity measurement. To determine the closeness of user relationships, it compares their relevance or similarity. Similarity measure is mainly calculated by similarity coefficient, correlation coefficient and different distances between feature vectors or attribute sets of objects, including cosine similarity coefficient, modified cosine similarity coefficient, Pearson correlation coefficient, Jaccard similarity coefficient and Euclidean distance (Singh, 2015; Zhang et al., 2015; Rao and Raju, 2016). On the one hand, the user-based collaborative filtering method requires more computational time, which becomes more un-pronounced when recommendation systems contain a large number of users and items. On the other hand, the disadvantage of the user-based method is that there are more parameters to be tuned.

It can be seen that if the Euclidean distance is greater, the similarity between users is smaller. When facing the users' score data with different dimensions and sparsity, some similarity measurement methods have some defects. For example, for excessively sparse user score data, when co-score projects are very rare, Pearson correlation coefficient is not good to measure similarity, and similarity calculation is not accurate.

Model-based collaborative filtering adopts users' historical score data to train a model and then make prediction based on the model. Common model-based collaborative filtering methods contain matrix decomposition and clustering. These traditional collaborative filtering methods are not applicable to the data recommendation under distributed systems, so this paper utilizes a modified LSH method to assist POI.

Currently, there are many researches focusing on user data privacy. Similarly, this paper also considers the users' data privacy on the platform. Homomorphic encryption is a common encryption method, which allows the computation on ciphertext for the purpose of privacy protection. (Li et al., 2016) proposed a privacy-preserving collaborative QoS (quality of service) prediction framework which could protect the private data of users while retaining the ability of generating accurate QoS prediction. It combined Yao's garbled circuit and additively homomorphic encryption via additively secret sharing to address non-linear computations required in the process of QoS prediction. Li and Rui (2018) improved matrix decomposition method and gave a new algorithm for protecting privacy based on non-negative matrix factorization and singular value decomposition. If using plurality kinds of decompositions, it could analyze data from different directions comprehensively. Therefore, it could find more non-critical information and improve the algorithm performance. Chen et al. (2017) proposed a protocol for conducting privacy-preserving ridge regression (PPRR) over high-dimensional data. In this protocol, each user submitted its data in an encrypted form to an evaluator and the evaluator computed a linear model of all users' data without learning their contents. The core encryption method was equipped with homomorphic properties to enable the evaluator to perform ridge regression over encrypted data.

Although these solutions provide privacy protection by secure multi-party computation protocol, the homomorphic encryption needs to consume a large amount of computing time and communication costs, and it cannot meet users' requirements for quick response. Therefore, using homomorphic encryption to encrypt all users' data on distributed systems cannot satisfy the practical needs of modern social applications.

Shoulin and Jie (2016) proposed a new social network privacy protection based on a new Map-Reduce model with a k-means approach. Main task controlled k-means to start iterative execution. Mapper sub-task independently computed the distance between each record and cluster center, then tagged them. Reducer sub-task calculated the sum of the record number in the same cluster and attributes vector. And it used the noise disturbance generated by Laplace. Liu et al. (2017) showed that accurate recommendation results could be obtained after adding random noise of specific distribution to original user data to prevent information leakage. Teng et al. (2018) presented an efficient and secure cipher-text retrieval scheme based on mixed homomorphic encryption and multi-attribute sorting method under cloud environment. This new scheme was divided into four steps: (1) Constructing multiple attribute characteristic vector safety index for documents; (2) Constructing reverse index for the uploaded documents and generating vector set of document, then computing module of each document vector; (3) Encrypting document vector set with homomorphic encryption and uploading them into cloud; (4) Adopting multiple attribute score formula to calculate document relevance score, according to the scores ranking to return the interesting retrieval results for the users. Guerraoui et al. (2015) presented a novel protocol distance-based differential privacy (D2P) that ensured a strong form of differential privacy, called distance-based differential privacy, and it was particularly well suited to recommenders. It combined random interference and differential privacy, a hybrid privacy protection recommendation system was proposed. The privacy of user data was protected by random interference, while the privacy of recommendation results was protected by differential privacy. Liao et al. (2018) developed the first Enhanced Privacy-built-In Client for Personalized Recommendation system that performed the data perturbation on the client side to protect users' privacy. It needed no assumption of trusted server and no change on the recommendation algorithms on the server side; and needed minimum user interaction in their preferred manner, which made this solution fit very well into real world practical use. However, in the social recommendation service, the efficiency is relatively low. So we propose a P^3^OI (privacy protection point of interest) recommendation algorithm based on multi-exploring locality sensitive hashing in this paper. The main objective of this work is to propose an effective solution to the POI recommendation problem in distributed systems. For this reason, we study a MELSH algorithm based on the concept of multiple exploration. The experiment on real data sets verifies that the algorithm can get the desired target better while ensuring the accuracy, confidentiality and efficiency of the recommendation algorithm.

The main contributions of this research are as follows:

1) We build offline user index and select MELSH function family. According to historical check-in data, the users on distributed platform are mapped to corresponding buckets.
2) Searching online similar users. According to the selected MELSH function family, the target user will be mapped to a bucket, where other users are considered to be similar to the target user in a high probability.
3) This paper introduces a multi-exploring method to improve the LSH algorithm. On the one hand, it reduces the number of hash tables to decrease the memory overhead; On the other hand, the retrieval range on each hash table is increased to reduce the time retrieval overhead.
4) Analysis shows that the proposed method has a better accuracy value compared with other methods.

## Proposed P^3^oi Recommendation Algorithm

Suppose that target *u*_1_ is a Weibo user, there is check-in data in location {*l*_1_, *l*_2_, ⋯, *l*_*n*_1__}; Target *u*_2_ is a Facebook user, there is check-in data in location (Shen and Jin, 2016) {*l*_1_, *l*_2_, ⋯, *l*_*n*_2__}; Target *u*_3_ is a Gowalla user, there is check-in data in location {*l*_1_, *l*_2_, ⋯, *l*_*n*_3__}. When using the user-based collaborative filtering method, the first step is to calculate the similarity between the target user and other users, but now the data are distributed on multiple platforms, which result in the following problems: (1) considering privacy issues of various platforms, data on Facebook and Gowalla will not be directly sent to Weibo platforms; (2) the amount of data on each platform may be very large, so it is very computationally intensive to calculate the similarity, which cannot meet the fast response requirements of target users.

To solve the above problems, we present a P^3^OI (privacy protection point of interest) recommendation algorithm based on multi-exploring locality sensitive hashing in this paper, it can better handle the recommendation problem under the distributed platform. First, some identifiers are defined.

1) *PF* = {*pf*_1_, *pf*_2_, ⋯, *pf*_*k*_} denotes *k* distributed platforms.
2) *U* = {*U*_1_, *U*_2_, ⋯, *U*_*k*_} is the user set corresponding to *k* distributed platforms. For *pf*_*k*_, its user set is *U*_*k*_ = {*U*_*k*_1__, *U*_*k*_2__, ⋯, *U*_*k*_*m*__}, m is the total number of users.
3) *L* = {*l*_1_, *l*_2_, ⋯, *l*_*n*_} denotes the n check-in locations. For the convenience of the discussion below, assume that check-in locations are the same for all platforms, namely, *n*_1_ = *n*_2_ = *n*_3_ = *n*.

### MELSH: Multi-Exploring LSH

LSH (Xia et al., 2015) is widely used in distributed systems. The basic idea of LSH is to closely link the possibility of conflict between two points with their distance, that is, the closer the two points are, the possibility of conflict is higher. Otherwise, it is lower.

Definition 1. Locality sensitive hashing (LSH). LSH relies on a family of hash functions, which are a set of map functions from point domain S to set domain D in space *R*^*d*^, expressed as *H* = {*h* : *S* → *D*}, randomly selecting a hash function *h*_*i*_(*i* = 1, 2, ⋯, *N*) to hash data points *a* and *b* in *R*^*d*^, if the following conditions are correct:

1) If ‖*a* − *b*‖ ≤ *r*_1_, so *p*[*h*(*a*) = *h*(*b*)] ≥ *p*_1_;
2) If ‖*a* − *b*‖ ≥ *r*_2_, so *p*[*h*(*a*) = *h*(*b*)] ≤ *p*_2_;

Where *p*[·] is probability function, 0 < *r*_1_ ≤ *r*_2_, 0 ≤ *p*_2_ < *p*_1_ ≤ 1. So the *h*_*i*_ is called (*r*_1_, *r*_2_, *p*_1_, *p*_2_)-location sensitive hash family.

**Figure 1:**
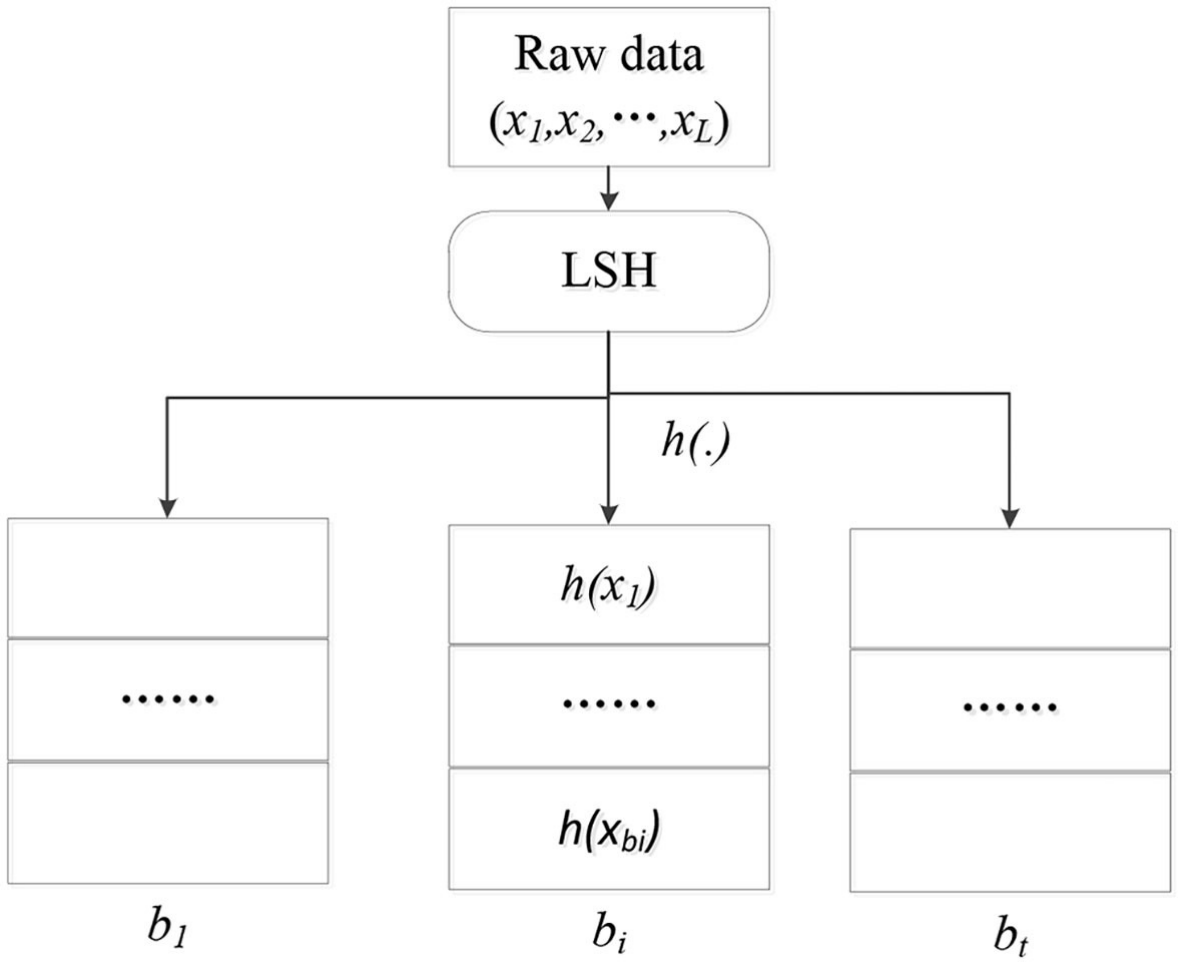
LSH principle.

As shown in Figure 1, the original *L* data points are mapped to *t* hash buckets by the hash function *h*(·). Each bucket contains a lot of adjacent data points, and the number of |*b*_*i*_| is much smaller than *L*, which also achieves the purpose of data dimension reduction.

If a target user wants to query data point similar to data point X. Then the point is mapped to the corresponding bucket by the hash function h(X), the data point in the bucket has a high probability with the similar data point of the user. Since the number of data points in the bucket is much smaller than *L*, the search efficiency is greatly improved. In addition, through the step of hash map, the privacy of users' original data can also be guaranteed, that is, users only know the hash value but do not know the original data of other users. Therefore, LSH can provide both efficient query and effective privacy protection in distributed platforms.

But there are two major problems with LSH: (1) it requires a large number of hash tables, each hash table is proportional to the size of the original data set. In particular, when the hash table takes up more space than the main memory size, there is a large amount of disk I/O for retrieval, causing serious latency and reducing time efficiency; (2) the query utilization rate on each hash table is not high, each time is limited to one bucket in the hash table for retrieval, that is, the retrieval value is equal to the hash value of the retrieval point, and the neighboring bucket of the retrieval point is ignored.

To solve the above problems, this paper introduces a multi-exploring method to improve the basic LSH algorithm. On the one hand, it can reduce the use of hash table to reduce the memory overhead; On the other hand, the retrieval range on each hash table is increased to reduce the time cost. Meanwhile, the retrieval quality is better than the original algorithm.

The core idea of multi-exploring LSH is to conduct multiple searches in each hash table, where the nearest neighbor point has a high probability of occurrence. It can explore all hash buckets that may contain the neighboring points of retrieval points. According to the property of locally sensitive hash function, if a similar target is “unfortunately” hashed into another bucket when it is close to the retrieval object, it is likely to be hashed into a bucket adjacent to the retrieval object. Therefore, the goal of multi-exploring LSH is to explore these adjacent buckets to find as many adjacent points as possible. The MELSH algorithm is described as follows:

For each d-dimensional data point *v*, hash data points *a* and *b*, the parameter ω in the hash function $h_{a,b}\left(v\right)=\left\lfloor \frac{a•v+b}{\omega }\right\rfloor$ is high under reasonable circumstance. In fact, it turns out that the hash values of all neighbors are approximate the same. Thus, the dimensionality of similarity points is reduced by the function $g_{i}\left(v\right)=\left[h_{1}^{i}\left(v\right),h_{2}^{i}\left(v\right),\cdots ,h_{k}^{i}\left(v\right)\right]\left(i=1,\cdots ,L\right)$ that can obtain k-dimensional vector *g*_*i*_(*v*). There may be a vector difference σ, and each component of this vector difference may be 0, 1 or 1. However, by exploring each adjacent vector *g*_*i*_(*v*) + σ, we can ensure that we can find more adjacent points of retrieve point and reduce the size of *L* at the same time.

### Paillier Homomorphic Encryption

Paillier is a difficult probabilistic public-key cryptography system based on composite decomposition. Paillier is an additive homomorphic encryption system. This paper uses Paillier to ensure that data information is not leaked in the calculation process. Homomorphism analysis of Paillier cryptography (Zhang et al., 2018) is as follows:

For Paillier: public key (*N, g*), private key (λ, μ).

1) Given a plaintext *m* ∈ (*Z*_*n*_), randomly selecting number $r\in Z_{n}^{*}$.
2) Encryption process: *c* ≡ *g*^*m*^ · *r*^*N*^ mod *N*^2^.
3) Decryption process: *m* ≡ *L*(*c*^λ^ mod *N*^2^) · μ mod *N*.
4) For plaintext *m*_1_ and *m*_2_ after encryption: $E\left(m_{1}\right)\equiv g^{m_{1}}x_{1}^{N}\left(\;\mathrm{mod}\;N^{2}\right)$ and $E\left(m_{\text{2}}\right)\equiv g^{m_{\text{2}}}x_{\text{2}}^{N}\left(\;\mathrm{mod}\;\;N^{2}\right)$.

We can obtain: $E\left(m_{1}\right)\cdot E\left(m_{2}\right)\equiv g^{m_{1}}x_{1}^{N}\cdot g^{m_{2}}x_{2}^{N}\;\mathrm{mod}\;N^{2}\equiv g^{m_{1}+m_{2}}{\left(x_{1}x_{2}\right)}^{N}\;\mathrm{mod}\;N^{2}\equiv E\left(m_{1}+m_{2}\right)$.

Therefore, Paillier public key cryptosystem satisfies additive homomorphism.

### P^3^OI Recommendation Algorithm

MELSH-based P^3^OI recommendation algorithm includes three parts:

1) Establishing offline user index, selecting MELSH function family. According to historical check-in data, the users on distributed platform are mapped to corresponding buckets.
2) Searching online similar users. According to the selected MELSH function family, the target user will be mapped to a bucket, where other users are considered to be similar to the target user in a high probability.
3) Recommending location of target users. Based on the similar user set obtained in the previous step, it uses the check-in data information of similar users to predict the target users' preference degree to some places without check-in, the recommended results can be obtained.

#### Establishing Offline User Index

It first selects a MELSH function *h*(*u*) or a family of MELSH function *H* = {*h*_1_(*u*), *h*_1_(*u*), ⋯, *h*_*r*_(*u*)} to build indexes for users distributed on different platforms. The selection of hash function is based on the distance definition 1. Since Pearson correlation coefficient is often used as a method to calculate similarity or measure distance in the recommendation system. This paper uses the MELSH function corresponding to Pearson correlation coefficient for index establishment.

For one user *u*, its historical check-in data can be expressed as a *n*-dimensional vector *u* = (α_*l*_1__, ⋯, α_*l*_*n*__), where α_*l*_*i*__ represents the check-in situation of the user. If α_*l*_*i*__ = 0, it indicates that the user did not visit *l*_*i*_. *v* = (*v*_1_, ⋯, *v*_*n*_) is a *n*-dimensional vector, where *v*_*i*_ is randomly selected from [−1, 1]. ○ denotes the dot product operation between vectors. The hash function is defined as follows:

(1) $$\begin{array}{l} h\left(u\right)=\left\{\begin{array}{c} 1,u○v>0\\ 0,u○v\leq 0 \end{array}\right\} \end{array}$$

By using the hash function, user *u* is hashed to a binary value of 0 or 1. MELSH is essentially a probability-based method, it can obtain more accurate similarity by introducing more hash functions or hash tables.

The proposed algorithm designed in this paper assumes that there are T MELSH tables, each table is composed of *r* MELSH functions. In each LSH table, each user *u* has a corresponding r-dimensional binary hash vector *H*(*u*) = (*h*_1_(*u*), *h*_2_(*u*), ⋯, *h*_*r*_(*u*)) after MELSH. Each value in *H*(*u*) is 0 or 1. If there is a hash table in *T* hash tables, let *u*_1_ and *u*_2_ be hashed to the same bucket after MELSH, then *u*_1_ and *u*_2_ are similar neighbors. In this way, we can index users on different platforms. In order to satisfy the requirements of quick response of users and improve the efficiency of searching, indexes of different platforms have service provider. According to the above analysis, the index established by MELSH will not expose the check-in data of platform users, so there is no privacy leakage problem.

#### Searching Online Similar Users

Based on the index of MELSH, target users who want to inquire similar users can use MELSH hash function to calculate the hash value according to their check-in records, and then find the corresponding similar users according to the hash value in the index stored in the service provider. All users in the bucket corresponding to the hash value are considered to be similar users.

#### Recommending Location of Target Users

This section describes the method that utilizes similar users to recommend POI to target users. To protect user data privacy on distributed platforms, user location check-in data is encrypted with Paillier and stored in the cloud with hash values. For location *l*_*tar*_ that the target user wants to predict, it can be calculated as follows:

(2) $$\begin{array}{l} \alpha_{l_{tar}}\;=\;\frac{1}{\left|SIM\right|}D\left(E\left(\underset{u_{i}\in SIM}{\sum }\alpha_{l_{tar^{i}}}\right)\right)\;=\;\frac{1}{\left|SIM\right|}D\left(\underset{u_{i}\in SIM}{\prod }E\left(\alpha_{l_{tar^{i}}}\right)\right) \end{array}$$

Where SIM represents the similar users set. |*SIM*| represents the number of similar users. $\alpha_{l_{tar^{i}}}$ denotes the check-in data at location *l*_*tar*_ of user *u*_*i*_. $E\left(\alpha_{l_{tar^{i}}}\right)$ represents the check-in data at location *l*_*tar*_ of user *u*_*i*_ with Pailler encryption. *D*(·) stands for Paillier decrypted. According to the property of homomorphic encryption, the number of visit by similar users to the predicted location is comprehensively considered, α_*l*_*tar*__ is the prediction result of location *l*_*tar*_.

MELSH-based privacy protection POI recommendation algorithm makes POI recommendation for target users by comprehensively utilizing users' check-in data on different distributed platforms, while protecting data privacy of each platform. The algorithm is described as follows:

**Algorithm 1:** MELSH-based P^3^OI recommendation.

**Input**. Distributed platform *PF* = {*pf*_1_, *pf*_2_, ⋯, *pf*_*k*_};
user set *U* = {*U*_1_, *U*_2_, ⋯, *U*_*k*_}. For *pf*_*k*_, its user set is *U*_*k*_ = {*U*_*k*_1__, *U*_*k*_2__, ⋯, *U*_*k*_*m*__}. Check-in location
*L* = {*l*_1_, *l*_2_, ⋯, *l*_*n*_}.
**Output**. Predicting results of *l*_*tar*_.
\\ Building offline user index
for t = 1 to T do // T hash tables
for i = 1 to k do // k distributed platforms
for j = 1 to m do
*H*_*t*_(*u*_*ij*_) = (*h*_*t*1_(*u*_*ij*_), *h*_*t*2_(*u*_*ij*_), ⋯, *h*_*tr*_(*u*_*ij*_));
for p = 1 to r do
for q = 1 to n do
*v*_*tpq*_ = random[-1,1];
end for
if *u*_*ij*_ ○ *v*_*tp*_ > 0
then *t*_*tp*_(*u*_*ij*_) = 1
else *h*_*tp*_(*u*_*ij*_) = 0
end for
end for
end for
\\ Searching similar users
Initializing SIM = empty set
for t = 1 to T do
*H*_*t*_(*u*) = [*h*_*t*1_(*u*), *h*_*t*2_(*u*), ⋯, *h*_*tr*_(*u*)];
for p = 1 to r do
if *u*_*ij*_ ○ *v*_*tp*_ > 0
then *t*_*tp*_(*u*_*ij*_) = 1
else *h*_*tp*_(*u*_*ij*_) = 0
end for
end for
end for
\\ according to *H*_*t*_(*u*), find corresponding bucket number, add similar users to SIM.
end for
\\ Target user location recommendation
tmp = E(0)
for i = 1 to |SIM| do
$tmp=tmp\cdot E\left(\alpha_{l_{tar^{i}}}\right)$
end for
$\alpha_{l_{tar}}=\frac{1}{\left|SIM\right|}D\left(tmp\right)$
return α_*l*_*tar*__.

## Experiments and Analysis

### Experiments Data

The experiment datasets adopt two public sets: film evaluation data set MovieLens provided by GroupLens in University of Minnesota (https://grouplens.org/datasets/movielens/) and social networking website Gowalla (http://snap.stanford.edu/data/loc-gowalla.html).

MovieLens includes 6,040 user votes for 3,706 movies, in total 106 grading records. Ratings are distributed in the range of [0,5]. If the rating is higher, then more users are interested in this movie consumingly. *T* denotes the number of hash tables, *r* is the number of hash functions. *m* is the number of users, *n* is the number of movies.

Gowalla contains 6,442,890 check-in data. Because the Gowalla data set is very sparse with a density of 2.08 × 10^−4^, the experiment in this paper extracts the check-in data of 10,000 users and corresponding 5,000 locations. The density of the experiment data set is 2.99 × 10^−3^. Where 90% of check-in data in the experimental data set is used as the training set, and 10% of the check-in data is used as the test set.

To measure the accuracy of P^3^OI prediction, root mean square error (RMSE) is used as the measurement standard. The accuracy of the prediction is measured by calculating the deviation between the predicted user check-in situation and the actual user check-in situation. Generally, the smaller the RMSE value is, the higher the accuracy of the prediction is. Suppose *qα*_*i*_ represents the predicted user check-in situation, α_*i*_ represents the actual user check-in record, *L*_*tar*_ represents the location set to be predicted, and |*L*_*tar*_| denotes the number of predicted site sets. The calculation formula of RMSE is as follows:

(3) $$\begin{array}{l} RMSE=\sqrt{\frac{\underset{i\in L_{tar}}{\sum }{\left(q\alpha_{i}-\alpha_{i}\right)}^{2}}{\left|L_{tar}\right|}} \end{array}$$

Another index is mean absolute error (MAE). The accuracy of the prediction is measured by calculating the deviation between the predicted user score and the actual user score. If the value of MAE is lower, then the recommendation quality is higher. Let the predicted user score vector be *p* = (*p*_1_, *p*_2_, ⋯, *p*_*t*_), the actual user score vector is *r* = (*r*_1_, *r*_2_, ⋯, *r*_*t*_). The MAE can be calculated as:

(4) $$\begin{array}{l} MAE=\frac{\overset{t}{\underset{i=1}{\sum }}|p_{i}-r_{i}|}{t} \end{array}$$

### Experiments Comparison

Experiments are carried out on the specific dataset. Firstly, according to the influence of parameter *k* on RMAE, the optimal parameters are trained, and then the recommendation quality and operation efficiency under different similarity measures are compared.

**Figure 2:**
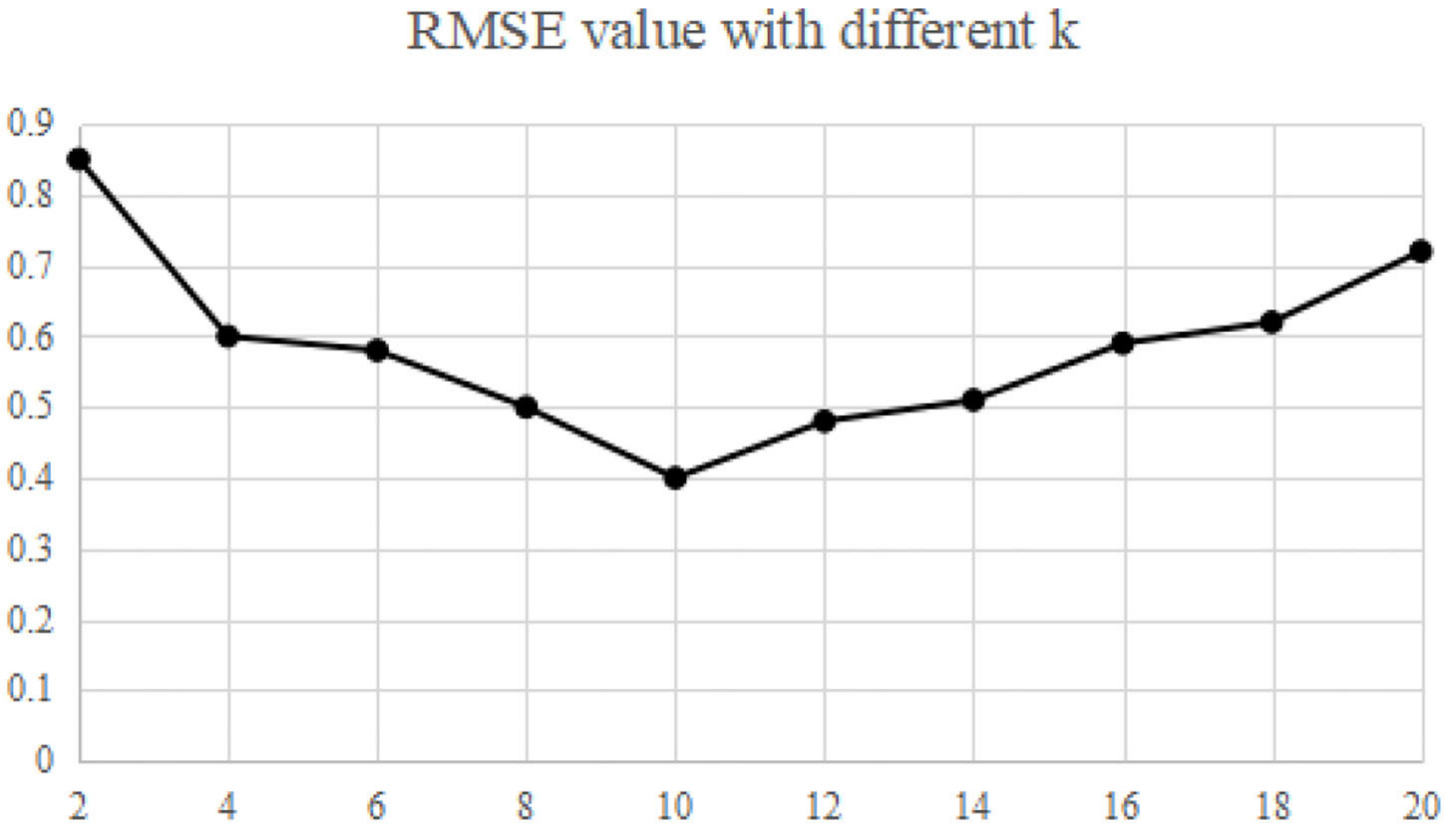
Impact of k on RMSE (MovieLens).

**Figure 3:**
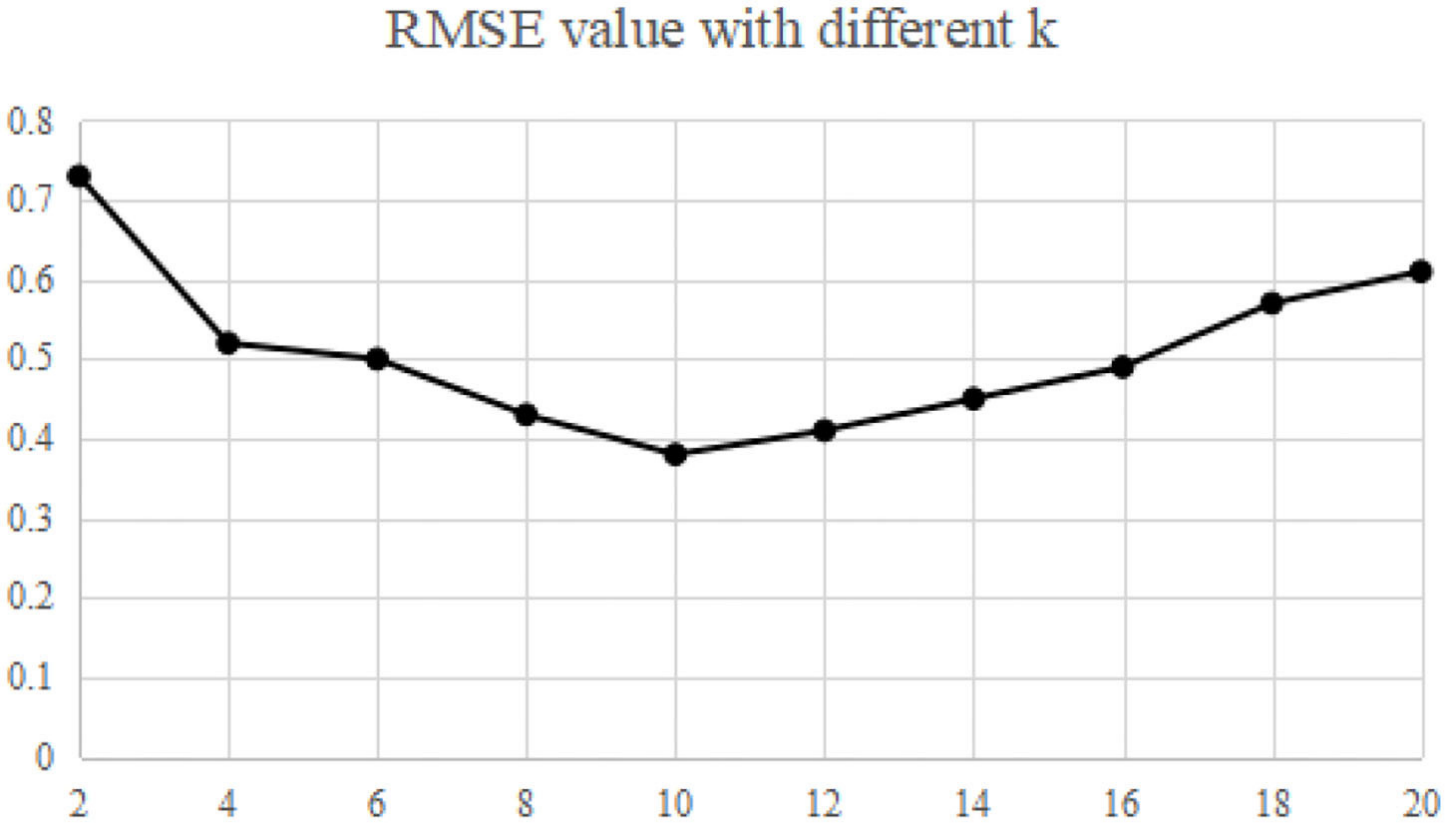
Impact of k on RMSE (Gowalla).

The recommended quality RMAE mainly depends on the key parameters *k* and *L* of MELSH algorithm. Considering the size of the data set and the actual running memory, the parameter *L* = 10 is selected in this paper. The choice of parameter *k* is trained according to the data set, and this parameter *k* is selected under the condition of ensuring the best recommended quality. The nearest neighbor number is set as 50, and the average RMAE is obtained through 10 experiments. We make experiments on MovieLens and Gowalla, the experimental results are shown in Figures 2, 3.

Experimental results show that when the parameter *k* = 10, RMSE is relatively the lowest, which is the best parameter in both experiments. More specifically, with this parameter, the recall rate and precision rate of similar users are relatively high and balanced to ensure high recommendation quality. If the parameter *k* is too small, false positive phenomena will exist in the neighbor users searching. If the parameter *k* is too large, it will cause “false negative” phenomenon. Both of the above phenomena will seriously affect the accuracy of neighbor user selection, and thus the accuracy of recommendation quality will be reduced.

We perform comparative experiments with the state-of-the-art recommendation methods including DTC (Liao et al., 2018), TSPOI (Wang et al., 2018), ABPR (Zhou et al., 2018) and our P^3^OI-MELSH on MovieLens and Gowalla dataset.

#### Experiment 1. Comparison for MovieLens Dataset

**Figure 4:**
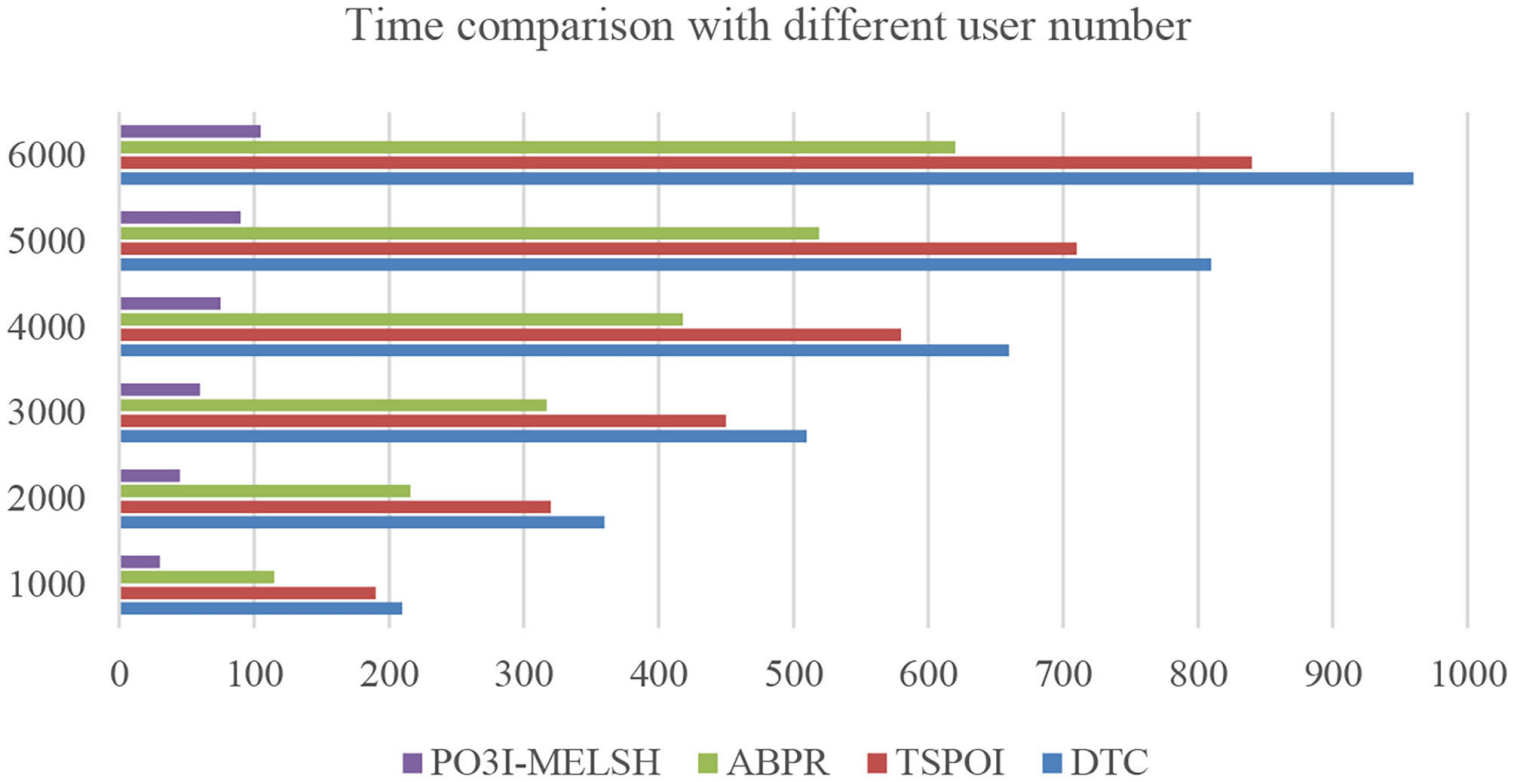
Effect of users number on time.

**Figure 5:**
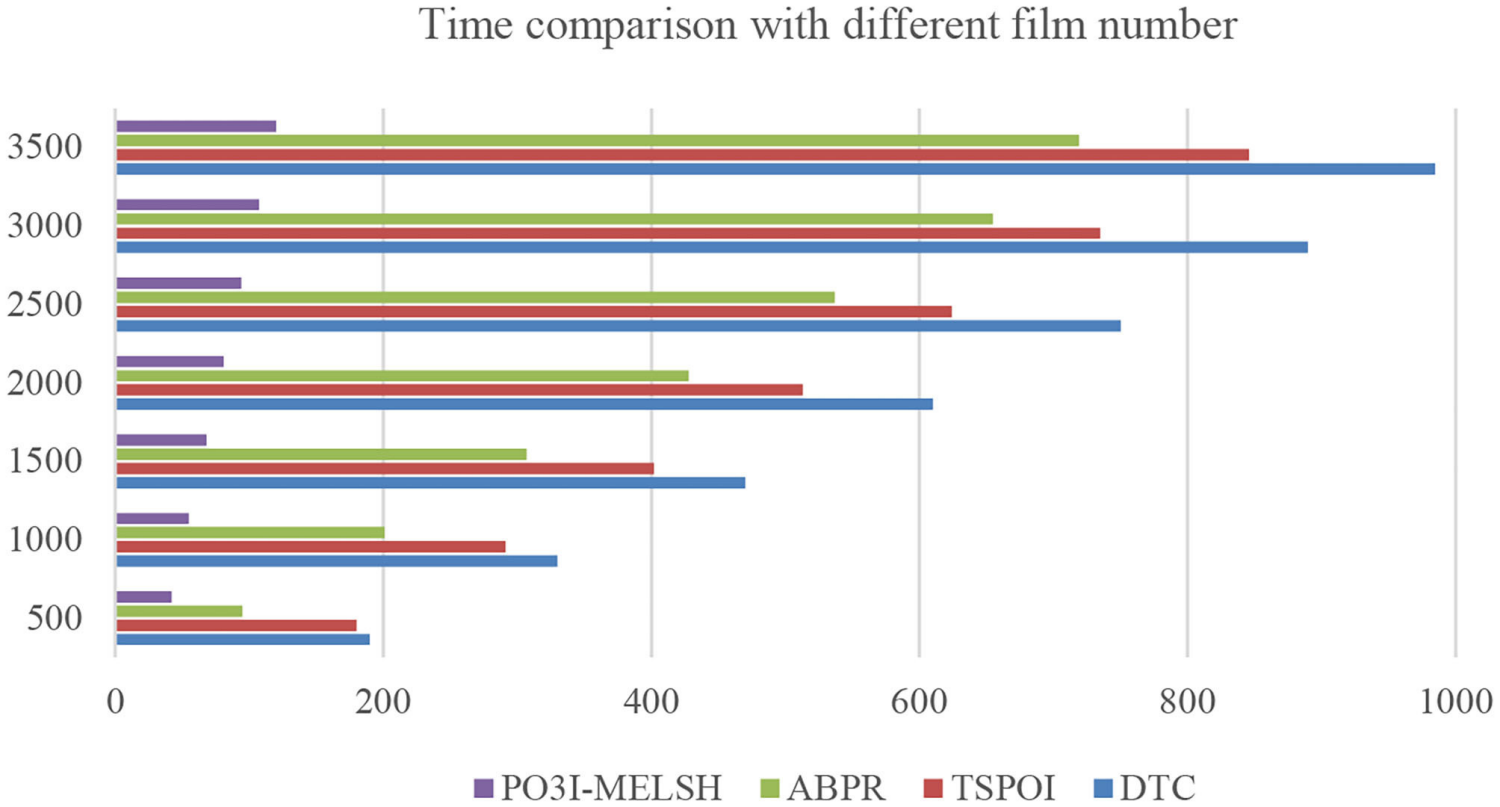
Effect of film number on time.

The efficiency comparison of the four recommendation algorithms with respect to user number and film number is shown in Figures 4, 5 respectively. Set T = 10, r = 10. In Figure 4, the number of users *m* changes from 1,000 to 6,000, and the number of films *n* = 3,500 remains unchanged. In Figure 5, the number of users *m* = 6,000 remains unchanged, while the number of films *n* varies from 500 to 3,500. Tables 1, 2 give the detailed measured value.

**Table 1:** Running time comparison with different user number (unit/s).

**User number**	**DTC**	**TSPOI**	**ABPR**	**P^**3**^OI-MELSH**
1 × 103	210	190	115	30
2 × 103	360	320	216	45
3 × 103	510	450	317	60
4 × 103	660	580	418	75
5 × 103	810	710	519	90
6 × 103	960	840	620	105

**Table 2:** Running time comparison with different film number (unit/s).

**User number**	**DTC**	**TSPOI**	**ABPR**	**P^**3**^OI-MELSH**
0.5 × 103	190	180	95	42
1 × 103	330	291	201	55
1.5 × 103	470	402	307	68
2 × 103	610	513	428	81
2.5 × 103	750	624	537	94
3 × 103	890	735	655	107
3.5 × 103	985	846	719	120

It can be seen from Figure 4, the running time of the four algorithms is increasing with the increase of m. However, P^3^OI-MELSH is still better than that of other three algorithms. The reason is that in P^3^OI-MELSH algorithm, according to the principle of MELSH, each check-in data is mapped to a hash bucket, the time complexity of P^3^OI-MELSH is O(m). The similar users in ABPR algorithm are more likely to visit the same locations. Therefore, the similarity between users will be calculated first, and then users with high similarity will be selected for recommendation. Therefore, the time complexity is O(m^2^). In Figure 4, m remains unchanged. As *n* gradually increasing, it can be seen that P^3^OI-MELSH is always superior to other methods. At this time, the time complexity of P^3^OI-MELSH is O(mn). Therefore, this algorithm has better collaborative filtering recommendation efficiency.

**Figure 6:**
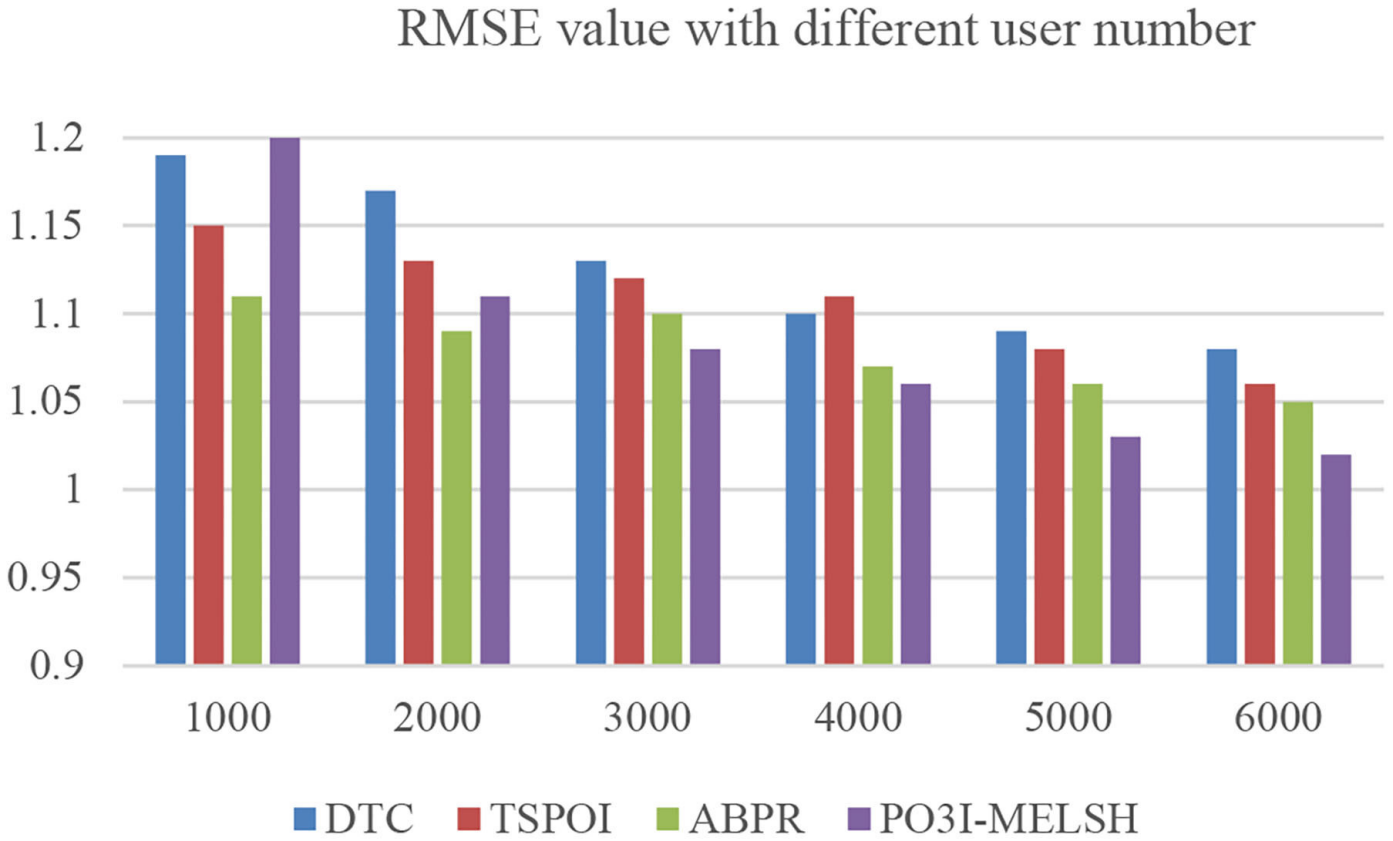
Effect of user number on RMSE.

**Figure 7:**
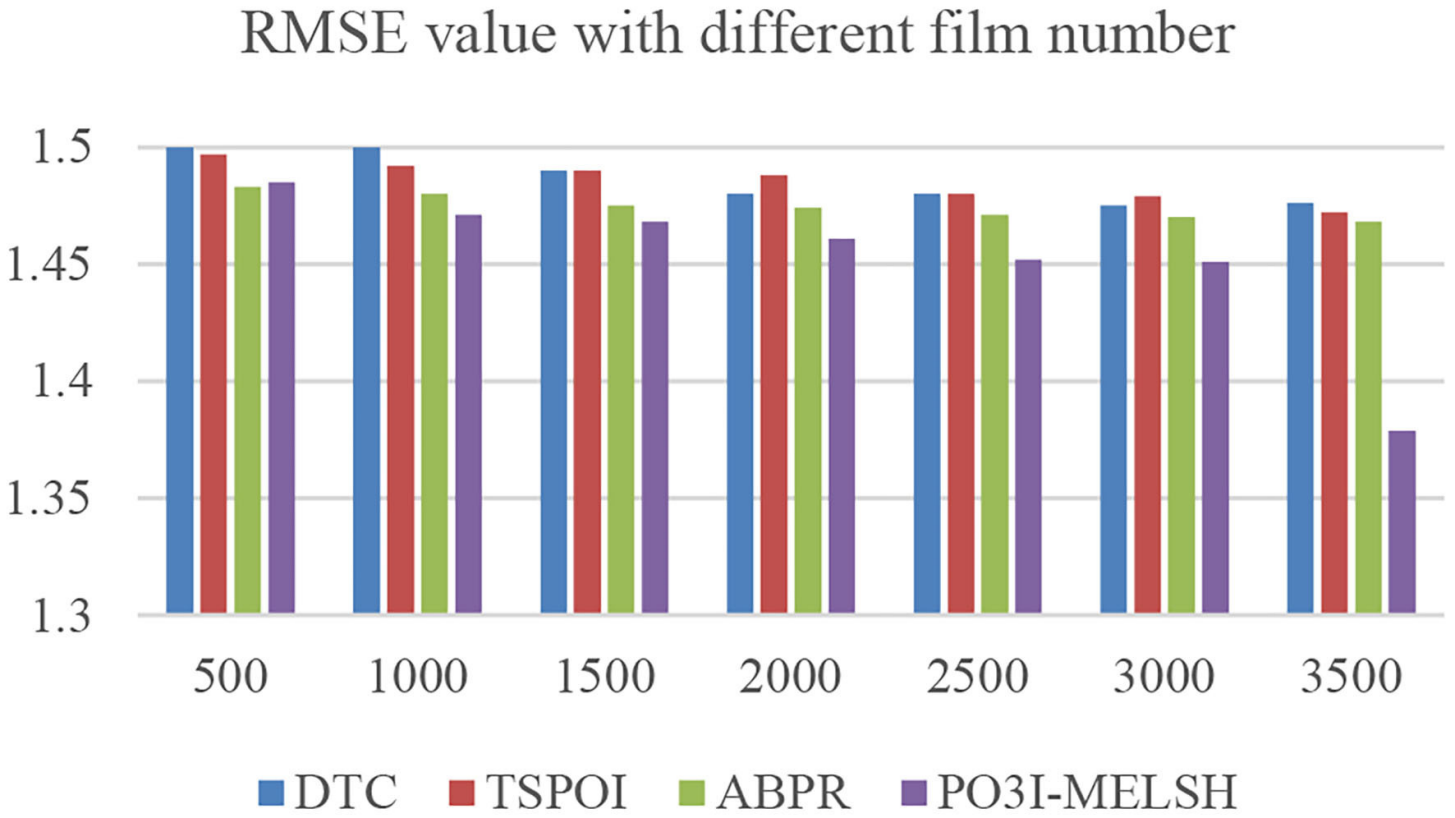
Effect of film number on RMSE.

The prediction accuracy comparison of the four recommendation algorithms with respect to user number and film number is shown in Figures 6, 7, respectively. Setting *T* = 10, *r* = 10. In Figure 6, the number of users *m* changes from 1,000 to 6,000, and the number of films *n* = 3,500 remains unchanged. In Figure 6, the number of users *m* = 6,000 remains unchanged, while the number of films *n* varies from 500 to 3,500.

#### Experiment 2. Comparison for Gowalla Dataset

**Figure 8:**
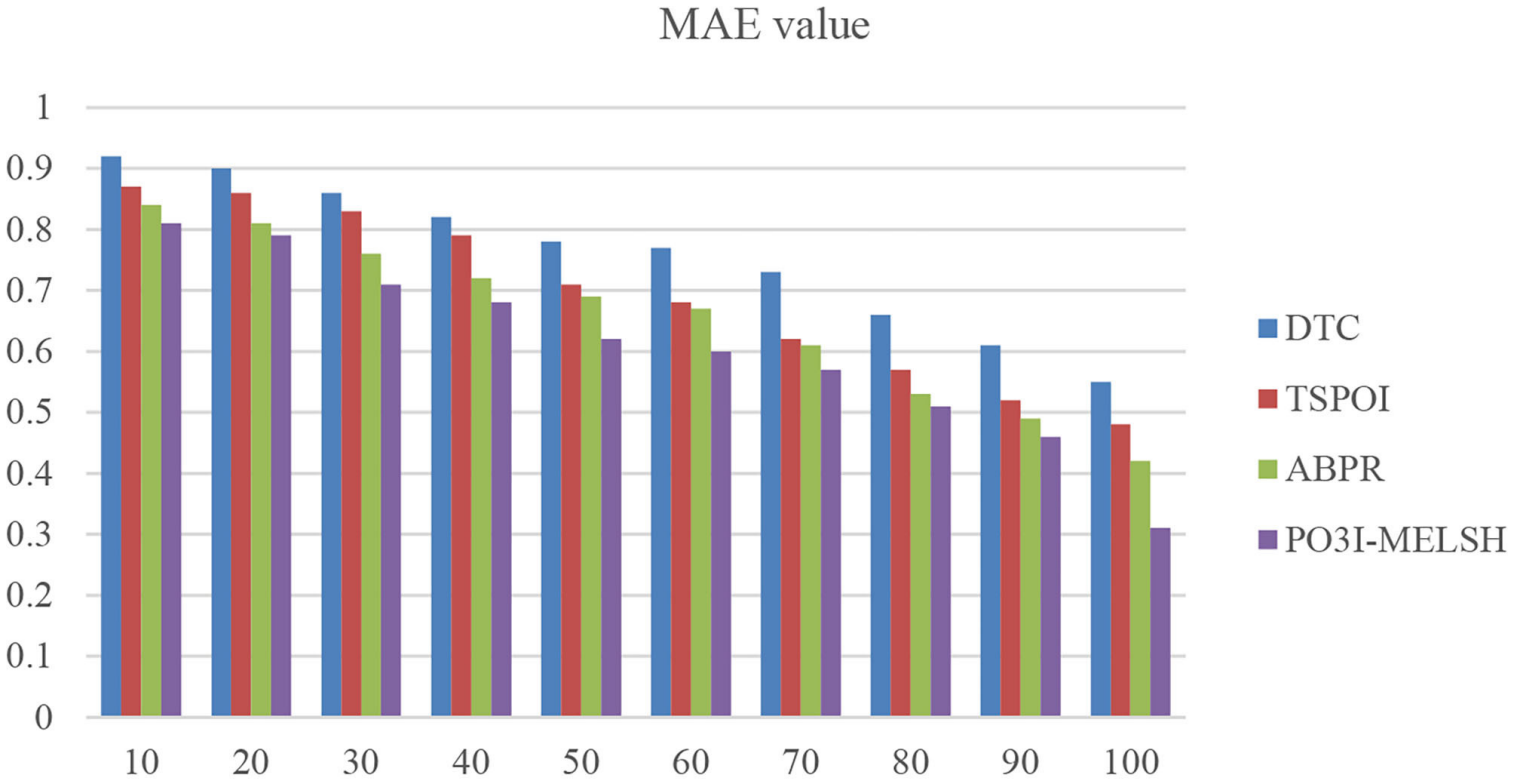
MAE comparison with different methods.

In the experiment, the nearest neighbor number of users is set as 10~100, parameter *k* = 12 and *L* = 10. The results are shown in Figure 8. Table 3 is the quantitative analysis.

**Table 3:** MAE comparison with different methods.

**Method**	**DTC**	**TSPOI**	**ABPR**	**P^**3**^OI-MELSH**
10	0.92	0.87	0.84	0.81
20	0.90	0.86	0.81	0.79
30	0.86	0.83	0.76	0.71
40	0.82	0.79	0.72	0.68
50	0.78	0.71	0.69	0.62
60	0.77	0.68	0.67	0.60
70	0.73	0.62	0.61	0.57
80	0.66	0.57	0.53	0.51
90	0.61	0.52	0.49	0.46
100	0.55	0.48	0.42	0.31

As shown in Figure 8, when there are fewer neighbor users, the MAE value of the P^3^OI-MELSH method is higher than that of the other methods. And as the number of neighbor users increases, the performance of P^3^OI-MELSH method is gradually higher than that of DTC, TSPOI, ABPR. It tends to be stable, which indicates the better effectiveness of the proposed method in this paper when the number of neighbor users is larger than a certain degree. At the same time, different number of users in the neighborhood will affect the recommendation performance. Therefore, the number of users in the neighborhood should be carefully selected in the actual recommendation system.

It can be further explained that MELSH random projection method prefers to select the nearest neighbor users with the same score, which is more accurate than the traditional method with directly calculating the similarity. Observing the experimental results of Figures 6, 7, we can see that under the premise of the same number of users and the same number of movies, the prediction result based on P3OI-MELSH is the most close to the actual situation. The experimental results in Figure 8 also prove this point.

**Figure 9:**
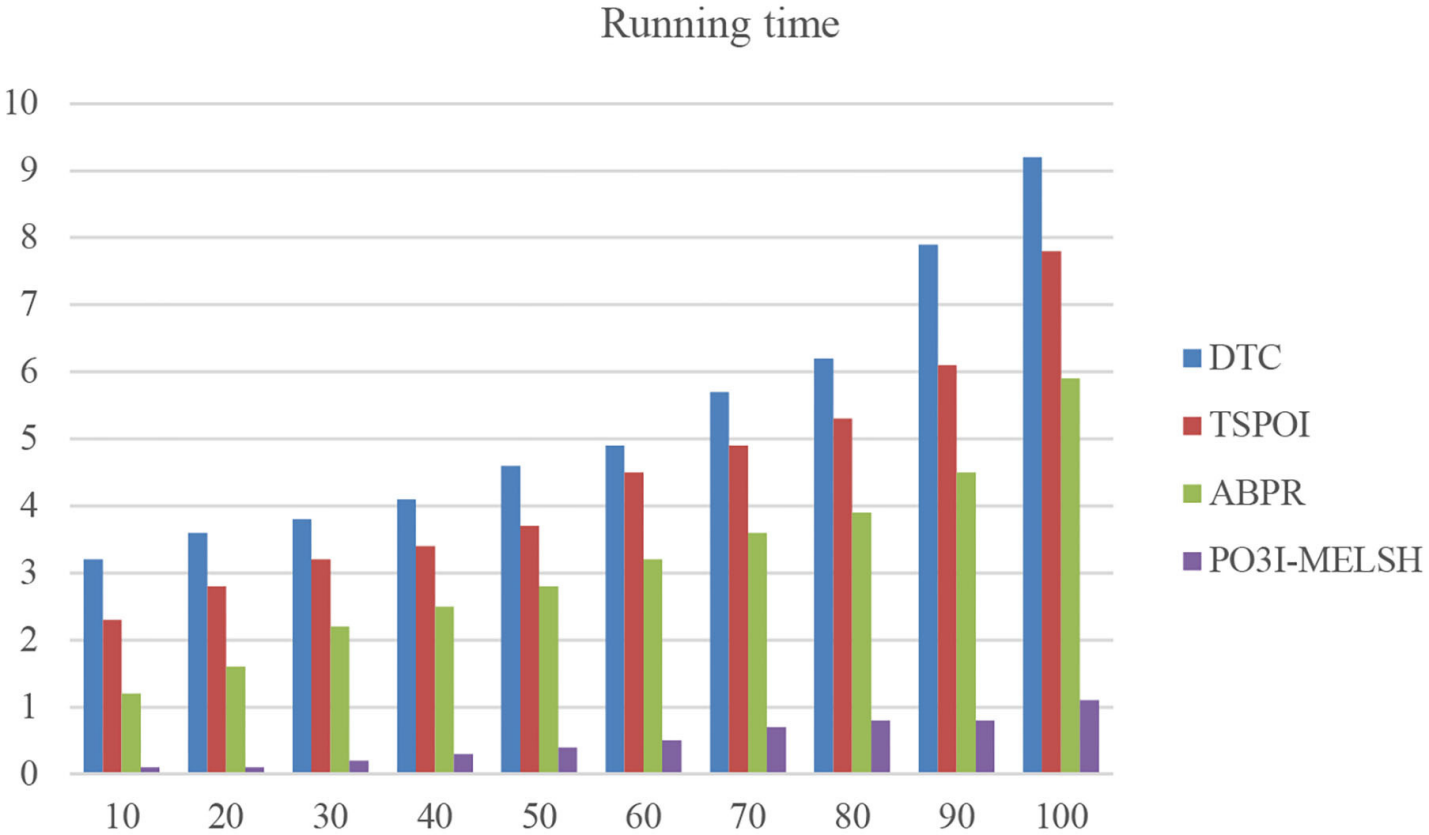
Running time comparison with different methods.

In order to verify the efficiency of the actual operation of the method in this paper, we select different number of neighbor users and conduct running time comparison with the other method as shown in Figure 9 and Table 4.

**Table 4:** Running time comparison with different methods (unit/ms).

**Method**	**DTC**	**TSPOI**	**ABPR**	**P^**3**^OI-MELSH**
10	3.2	2.3	1.2	0.1
20	3.6	2.8	1.6	0.1
30	3.8	3.2	2.2	0.2
40	4.1	3.4	2.5	0.3
50	4.6	3.7	2.8	0.4
60	4.9	4.5	3.2	0.5
70	5.7	4.9	3.6	0.7
80	6.2	5.3	3.9	0.8
90	7.9	6.1	4.5	0.8
100	9.2	7.8	5.9	1.1

As shown in Figure 9, the running time of other methods increases sharply with the increase of neighbor users number, while the overall running time of P^3^OI-MELSH is relatively stable. New method's efficiency is several times higher than that of the DTC, TSPOI, ABPR method. This proves that the locally sensitive hash algorithm is efficient and stable, suits for different scales data. The similarity retrieval is completed in linear time.

Overall, P^3^OI-MELSH has good prediction effect in most cases. The increasing of user number in both methods leads to the decline of prediction accuracy. The reason is that the real data set used in this paper is relatively sparse. When the number of users increases, noise data is introduced, which leads to the decline of prediction accuracy.

Taking time consumption and prediction accuracy into consideration, the algorithm proposed in this paper is not only able to respond to the user requirements quickly, but also provide better prediction results.

**Figure 10:**
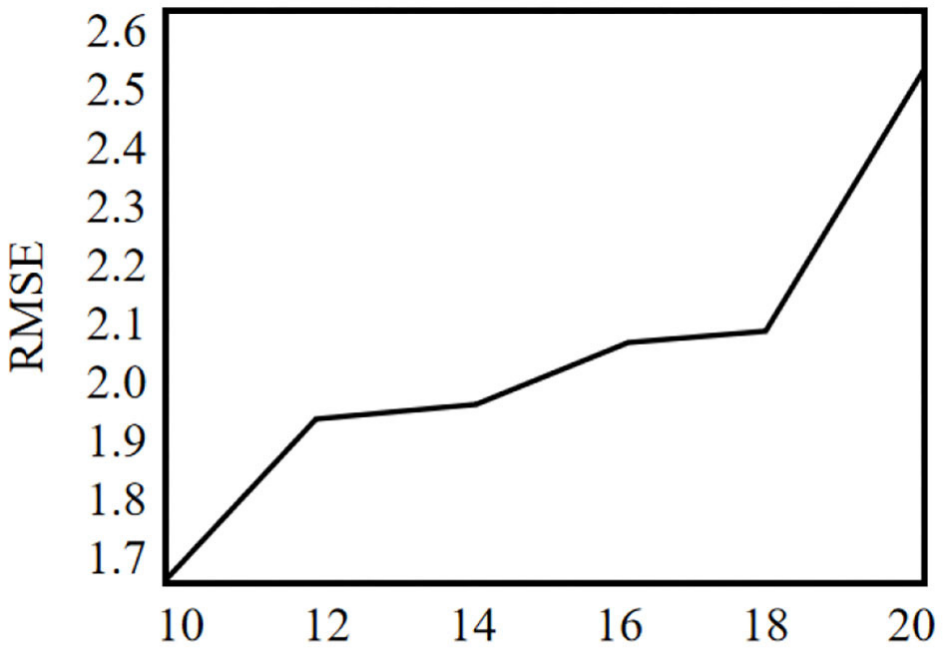
The effect of hash tables number on the RMSE value.

**Figure 11:**
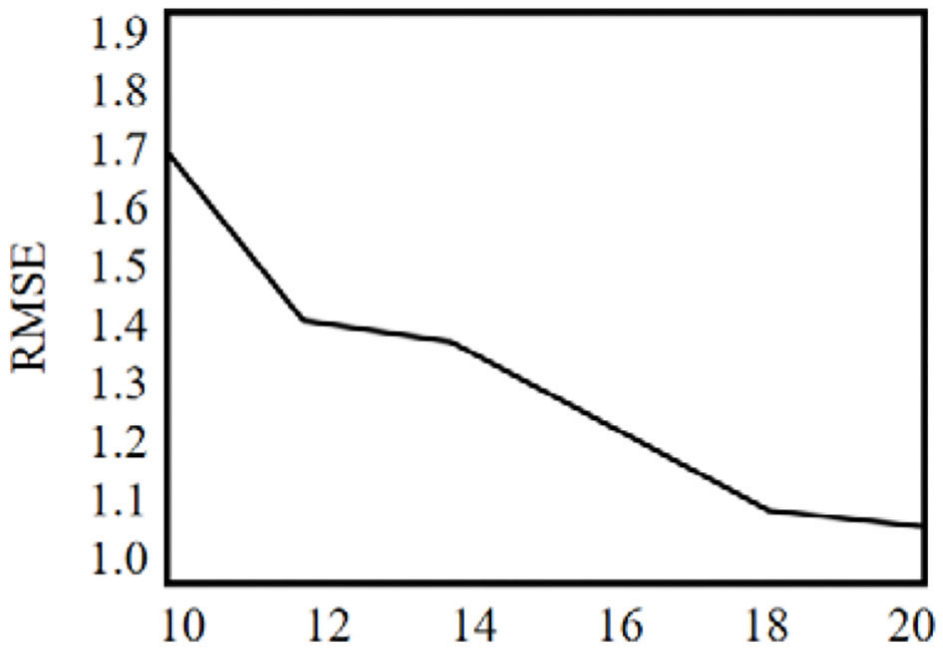
The effect of hash functions number on the RMSE value.

P^3^OI-MELSH algorithm is a privacy protection POI recommendation algorithm. The number of hash table and hash function in MELSH has an important impact on the mapping of user check-in data to hash hash value. Thus that will affect similar user sets. This section tests the influence of different *T* and *r* on prediction accuracy, and the experimental results are shown in Figures 10, 11. Set *m* = 6,000 and *n* = 3,500. The number of hash table T in Figure 10 varies from 10 to 20, and the number of hash function *r* = 10 remains unchanged. In Figure 11, the number of hash table *T* = 10 remains unchanged, and the number of hash functions n varies from 10 to 20.

It can be seen from the Figure 10, with the increase of hash table number, the prediction accuracy of P^3^OI-MELSH algorithm is declined, because similar user set SIM chooses union of similar user sets in different hash tables. With the increase of *T*, some users may also enter the collection not similar to SIM, and these noise data affect the accuracy of the prediction. To improve the prediction accuracy, in the practical application, it should choose a smaller hash table number.

It can be seen from the Figure 11, with the increase of hash function number, prediction accuracy of P^3^OI-MELSH algorithm is improved. The reason is that in algorithm design, with the increase of *r*, hash function design is more strict. Only very similar users can be mapped to a hash bucket to form a similar user set SIM, which guarantees the accuracy of the recommendation results based on these high probability of similar users. Therefore, in order to improve the accuracy of prediction, a larger number of hash functions should be selected in practical applications.

## Conclusion

In this paper, we use the MELSH and Paillier encryption to protect data privacy in different social platforms, and provide better prediction results. Experimental results show the efficiency and effectiveness of the proposed algorithm in this paper. The response time and prediction accuracy are superior to the traditional collaborative filtering recommendation algorithm based on the user and other state-of-the-art methods. In the future, we will research more advanced point of interest recommendation algorithms to improve the prediction accuracy.

## Data Availability Statement

The data analyzed in this study is subject to the following licenses/restrictions: The data used to support the findings of this study are available from Shoulin Yin. Requests to access these datasets should be directed to Shoulin Yin (yslinhit@163.com).

## Author Contributions

DL, LS, and CW: drafting and refining the manuscript. SY and HW: critical reading of the manuscript. All of the authors have read and approved the manuscript.

## Conflict of Interest

The authors declare that the research was conducted in the absence of any commercial or financial relationships that could be construed as a potential conflict of interest.
